# Consent as a compositional act – a framework that provides clarity for the retention and use of data

**DOI:** 10.1186/s13010-024-00152-0

**Published:** 2024-03-06

**Authors:** Minerva C. Rivas Velarde, Christian Lovis, Marcello Ienca, Caroline. Samer, Samia Hurst

**Affiliations:** 1 Geneva School of Health Science, University of Applied Sciences Geneva HES-SO, Geneva, Switzerland; 2 Division of Medical Information Sciences, Department of Radiology and Medical Informatics, University Hospital of Geneva, Geneva, Switzerland; 3 Institute for Ethics and History of Medicine, Department of Clinical Medicine, School of Medicine and Health, Technical University of Munich, Munich, Germany; 4 Division of Clinical Pharmacology and Toxicology, Geneva University Hospitals, Geneva, Switzerland; 5 Institute for Ethics, History, and the Humanities (iEH2), Faculty of Medicine, University of Geneva, Geneva, Switzerland; 6 College of Humanities, Swiss Federal Institute of Technology in Lausanne, Lausanne, Switzerland

**Keywords:** Informed consent, Dynamic consent, Framework, Information Technology, Trust

## Abstract

**Background:**

Informed consent is one of the key principles of conducting research involving humans. When research participants give consent, they perform an act in which they utter, write or otherwise provide an authorisation to somebody to do something. This paper proposes a new understanding of the informed consent as a compositional act. This conceptualisation departs from a modular conceptualisation of informed consent procedures.

**Methods:**

This paper is a conceptual analysis that explores what consent is and what it does or does not do. It presents a framework that explores the basic elements of consent and breaks it down into its component parts. It analyses the consent act by first identifying its basic elements, namely: a) data subjects or legal representative that provides the authorisation of consent; b) a specific thing that is being consented to; and c) specific agent(s) to whom the consent is given.

**Results:**

This paper presents a framework that explores the basic elements of consent and breaks it down into its component parts. It goes beyond only providing choices to potential research participants; it explains the rationale of those choices or consenting acts that are taking place when speaking or writing an authorisation to do something to somebody.

**Conclusions:**

We argue that by clearly differentiating the goals, the procedures of implementation, and what is being done or undone when one consent, one can better face the challenges of contemporary data-intensive biomedical research, particularly regarding the retention and use of data. Conceptualising consent as a compositional act enhances more efficient communication and accountability and, therefore, could enable more trustworthy acts of consent in biomedical science.

## Background

Informed consent is one of the key principles of ethically appropriate research involving human participants. When research participants give consent, they perform an act in which they utter, write or otherwise provide authorisation to somebody to do something. The goals, validity of procedures, and purposes of informed consent have been extensively explored in the bioethics literature [1–3]. However, do the goals and procedures presuppose the meaning of consent? Or, is the act of consenting to be explained in terms of what it does or undoes? This paper proposes a new understanding of the informed consent mechanism as a compositional act, moving away from a modular conceptualisation of informed consent procedures. We propose a formalisation of the composition of the intention of informed consent as a compositional act.

This paper presents a conceptual analysis of consent acts with the aim of exploring what consent is and what it does or does not do. First, we lay out the difference between the goals of informed consent and the required steps of informed consent procedures. We then present informed consent as a compositional act. For that purpose, we break down the notion of consent into its constitutive parts and subsequently present a framework which formalises the composition of the informed consent act. We further analyse the consent act by first identifying its basic elements, namely: (a) specific agents -data subjects or legal representatives- that provide the authorisation of consent; (b) a specific thing that is being consented to; and (c) specific agent(s) to whom the consent is given. We then explore the characteristics of each of them, their own separate purposes and their interaction that results in a consent act. We conclude by addressing the limitations and further lines of inquiry arisen from the afore-delineated framework. We argue that, by explaining what consent does or undoes, one can enhance the trustworthiness of the acts of consent in the biomedical sciences and medicine.

## Method: conceptual analysis of goals, processes and what informed consent procedures ought to be

### The goals of informed consent

In research with human participants, informed consent aims to protect the rights and duties of participants [1–3]. Its core purpose is to enhance participant control over effective authorisation limiting deception and minimising coercion [4]. Mason and O’Neil [5] describe informed consent as a waiver of normative expectations. This means that informed consent validation shall assess the context and norms of legitimate expectations and outline where this expectation might be transgressed. Informed consent builds upon effective communication between research agents and research participants, in which the research participant is provided with accessible and relevant information [5]. This conceptualisation follows a robust history of the theoretical accounts of how informed consent as a process ought to be, which we now briefly outline.

### Informed consent as a procedure

The work of Beauchamp and Childress [3] in the conceptualisation of consent as a philosophical, legal, and policy requirement procedure for health research continues to be a reference point and is often regarded as the traditional view of consent. For them, informed consent is composed of five building blocks that start with (a) disclosure of relevant information for potential participants to judge risk and benefits, followed by (b) effective comprehension of this information by the research subject; (c) voluntariness, the choice to consent must be freely made, (d) competence which refers to decision-making capacity regarding the presented choice, and (e) consent. Together they are ‘legally or institutionally effective authorisation from a patient or subject’ (P. 280). These concepts are seen as basic elements of procedures, thus sometimes referred to as pre-requirement of informed consent [6]. A key feature of Beauchamp’s and Childress’ [3] conceptualisation is to portray informed consent procedures as an opportunity for a substantially autonomous choice by the research participant about whether she/he authorises something such as medical intervention or participation in research.

Later, Beauchamp added a more refined and modular perspective to his early notion and argued for a modular approach to informed consent [7]. He claimed first that competence is a threshold element that stands apart from the other element and is a precondition of informed consent. This reinforces the notion of the informed consent process. Then, he also argues that disclosure is unnecessary if the person already has relevant information. Furthermore, he reiterates that consent is both a decision in favour of a proposed course of action and an authorisation. Also, outline a distinction between two different meanings of informed consent. The first informed consent is an individual’s *autonomous authorisation* of medical intervention or participation in research. The second one, informed consent, is an applied set of ‘social rules of consent that determine legally or institutionally valid consent’ Beauchamp, P 58. Nevertheless, Thus, even on this second case informed consent ought to serve as criteria for the moral adequacy of a given institutional rules. In our approach we lay towards the second meaning of consent.

### How informed consent procedures ought to be

Authorisation or consent is presented as a static action based on building blocks - disclosure, comprehension, voluntariness, and competence- if those are not met, informed consent is deemed invalid. The operationalisation of the procedure of informed consent has often fallen short [8, 9]. Manson and O’Neill [5] recalled that the goal of consent is to limit deception and coercion and to enhance control over the amount of information participants or patients receive, as well as, the opportunity to revoke consent already given. Thus, in the Beauchamp and Childress model the potential research participants/patients do not have any control over disclosure of information and, consequently, to what they are consenting to.

O’Neill critics highlight a key point, control over the amount of informati*on received and to what exactly is consented or not*. This point is becoming increasingly important. Contemporary literature explains that traditional views on the consent process and its philosophical premises are inadequate for big data, especially data-intensive medical research such as genomic research [10–13].

Biomedical research and informed consent procedures have been transformed through the application of information technologies such as pervasive and ubiquitous computing, digital health applications and advanced machine learning algorithms for data analytics. Over the last decade, alternative models of informed consent have emerged that aim to address the complexities of data-intensive research. These models portray consent as the result of the continuous interaction between researchers and participants [14]. One of these is broad consent, which requires investigators to offer research participants a range of options in relation to consent to the ongoing storage and future use of their personally identifiable data. This idea has proven particularly useful for biobanks and has been incorporated into regulation policies around the globe [15–17]. However, broad consent presents the challenging task of informing research participants about potential future (re)uses of data collected and it is difficult to say that such an approach would provide a sufficient amount of information specification necessary for the participant to reasonably provide informed consent [18]. Controversies in these models, however, have arisen from the extent to which the personal preferences and autonomy of research participants are respected and the extent to which research participants are actually informed about the research.

Other authors [14, 19] thus proposed to move away from static decision-making via a digital communication interface that connects researchers and participants, favouring a dynamic choice and enhancing a subject’s control over their preferences. This information and communication web-mediated model proposes a dynamic platform that facilitates bidirectional communication in which potential research participants can modify and manage their own consent preferences, often, as much as they wish. Similarly, collaborative consent [20] appeals to the same logic of using an IT digital communication interface, distinguishing from the former by focusing on the dialogue between the potential research participant and the research entity. Collaborative consent portrays consent as a collaborative process between the individual and the enterprise. A third approach comes from portable legal consent [21] which aims to directly address the challenges of genomic research. Portable legal consent tries to create a shared, open-source repository of data in which participants can tailor their preferences via a digital platform. Participants donate their data to the database under the promise that data will be used ethically and their privacy will be protected. All of these approaches are worthy of further testing and many questions remain unanswered. Furthermore, these models show a growing evidence that Beauchamp’s and Childress’ [3] conception of the notion of autonomous choice, which is at the heart of their traditional model of informed consent, is unfit for informed consent procedures via digital interfaces. This is due to the following reasons: firstly, the potential research participants cannot gain a substantial degree of understanding of every decision task they might be consenting to due to the vast amount of information that this might imply, a problem known as ‘informational overload’. Secondly, the freedom of the individual is constrained by the systems’ operability and procedural transparency [22, 23]. Thirdly, the assessment of competence or incompetence is particularly problematic, at times discriminatory and often not feasible [24]. But, beyond the poor fit of traditional models of consent to interactive online dynamic platforms, as potential research participants are provided with an increasingly broad spectrum of choices, further reflection is lacking on what choices are to be made and why those and not others? What choices result in informed consent? Widening the range of choices without a sound reflection of their meaning would not be conducive to trustworthy consent procedures either. Anker et al. [25] found that increasing the range of choices within informed consent interfaces makes little differences to individuals if they do not trust either the tool or the research actor(s) behind it. Trust between potential research participants and research actors is fundamental in informed consent, as well as for the development of consent models [25, 26].

Furthermore, a recurring issue across these models is their assumption that informed consent must be sought and given in a uniform manner for everything and everyone. Mason and O’Neil [5] claimed that such a view is ethically wrong, pointing out that not all research participants and/or patients want to be burdened with vast amounts of information and details, while others might need a deeper understanding. These models do explain the gains offered by interactive models of consent regarding the possibility to tailor and update consent to one’s preference, but not enough attention has been paid to the potential nudging of participants’ choices linked to web-design. Although people have a reasonable chance to be informed and to voluntarily choose, options will be limited to both available information and system functionality [23]. Furthermore, there is little attention paid to data recipient(s) and their obligation to the data subject and rightful use of acquired data. Thus, the sharing and re-sharing of data is becoming a key feature of the health and research landscape. This also brings up issues of data storage and re-use of data as usage and research purposes evolve, as knowledge progresses it is often unclear to research practitioners if they do or do not have valid consent to re-use personalised data that has been archived. Moreover, the role of revoking consent in the continuum of the informed consent process is hardly mentioned.

To address the shortcomings outlined above, Manson and O’Neill [5] provides a more concrete conceptualisation of consent. In their view, consent is an act resulting from rationally evaluable social transactions between agents in which consent is sought, given or withheld. Agents are judged on their capacity to do something in a competent, reliable, and honest manner. Therefore, informed consent is given to a specific agent to perform a specific act in a competent, reliable, and honest manner. This act is based upon intelligent trust [27], which is the results of active enquiry and trustworthiness.

Trust is highly situational [28]. In terms of digital consent, trust that one actor will perform a specific act in a competent, reliable, and honest manner will be influenced not only by the traits of the actor and institutions seeking consent but by various factors such as the digital platform, legal frameworks and culture, to name a few. Trust and trustworthiness are related concepts. Trustworthiness claims Jones lies in the expectation and the demands imposed by others and is not up to the actor [29]. For example, trustworthiness on an AI-powered tool may not always lead to trust [28]. O’Neill state ‘Trust is valuable when placed in trustworthy agents and activities’ [27] P.293. In the context of consent, we trust an actor lets say researchers, to do something in a reliable, honest and competent manner. The action authorised shall also be worthy of trustworthiness and open to scrutiny.

This view of consent as a communicative act can be taken further [30]. Taking this idea forward, we believe that focusing on what consent does or undoes is a better way to address the purpose of informed consent, which is limiting deception and coercion and enhancing individual self-determination.

## Results from conceptual analysis: properties of informed consent

Based on Manson and O’Neill’s views, consent is an act in which a person gives consent to a specific agent to perform a specific act. We propose a formalisation of the compositional elements of consent and their intention. Consent has three indivisible elements: (a) specific agents -data subjects or legal representatives- who provide the authorisation of consent; (b) a specific thing that is being consented to; and (c) specific agent(s) to whom the consent is given. Each of these elements has similar or dissimilar characteristics, thus, each has its own separate purpose, and neither is the same. As in a periodic table, by looking at each element and its characteristics, we can tell what acts might result from combining them. For example, we can tell if a data subject’s characteristics such as age, cognitive function, ethnicity, legal status or alike might require to tailor consent procedures or impact on the adequacy of the second element. The second element is the ‘specific thing that is being consented to’, such as, approving or denying access to medical files; participation in a trial for a new drug; or donating DNA for research, is also impacted by the third. The third ‘specific agent(s)’ giving consent to a public university lab, government facility that discriminates against a social group such as the lesbian, gay, bisexual, transgender and intersex (LGBTI) community or to a profit-oriented enterprise based offshore might show some different property-based dynamics, and therefore the resulting act.

Table 1 illustrates these points and adds further elements that are arranged in blocks or in groups with similar properties. The table lines and columns go from specific to general; in which we can select one or more compositional elements per line and per column that together will result in a concrete act, a specific compositional act of consent. For example, data subjects can be average adults; minors; legal representatives; collectivities; and those who may require tailored protection such as those under government custody, minorities or persons with difficulties processing information. Block 2, addresses what is being consented to. It covers different elements that compose the action of consent, including what exactly is being consented to and under which conditions one would allow profiting from data, as well as timeframes. Finally, the last block outlines some common recipients of consent, including official collective authorities. The latter may be called differently in different parts of the world, and they often exist in various forms, and while they are not always present, they very often are.

For example, an adult consent to donate his/her health data, as well as social context data, tissue and to actively participate in this and further research as long as profit is not sought until he/she revokes in this or any jurisdictions for research that aims to improve health advice, care treatment for him/her and other, will generate public benefits and progress in science. Thus, this consent is given only to health actors under official collective authorities (often the public hospital) and not to be shared with other official collective authorities like the policy, school system or the, tax office or private enterprises.

**Table 1:** Elements of informed consent

**data subject**		A person over 18 years	Person under 18 years	legal representative	collective	Person under government custody	Person who self-identify as ethic minority	Person with difficulties processing information
**Consent to**	Goals	To advance science	To do research in any area	Improving health systems	For research, public benefit		For profit-driven research	
	Action	Donate my health data	Donate social context data	Donate tissue and extracted data from my tissue	Donate complementary data on this and future clinical research	Donate complementary data to this and future qualitative research	Donate all my data for secondary research use	
	Benefits	For research, public benefit	To receive tailored advice	To improve care for me	To do disease research on the illnesses, I live with.	To improve care for others	To do research outside of health	For self-profit
	Timeframes	Indefinitely	As long as I am alive	Until my next visit to a health setting	Until I revoke	As long as legislation does not change	for 5 years	Today
	Risk	Low		Medium		Hight		
	Jurisdictions	Any jurisdiction	Supra national political organisation	Country of legal residence	Country of citizenship	Territories with the same or more protections	Jurisdiction with the same legislation within this country	This county
**Agents**		Health actors under Official collective authorities	under Official collective authorities	Private Education institutions	Any Official collective authorities	Private companies	Commons	Everybody

## Discussion of the elements of consent

### Data subject

Data subjects or legal representatives providing the authorisation of consent to somebody. A data subject is an identifiable natural person [31]. This concept borrows from current legislation. It implies that persons may be identifiable by name, email address or an online identification. This table includes some broad categories that influence weather or not a person could provide consent. i.e. legal age. Thus, it also includes other broad categories that are linked to social and legal features that may define whether a person can give consent. This list is by no means exhaustive. This list is limited to some broad categories of vulnerable individuals that have been exposed to inherent or intended risks or may need guarantees that their interests are safeguarded. It’s important to keep it broad, perhaps linking to existing legislation directed to protect specific group [32]. There will be little value of adding up every personal feature.

### Specific actions

This component outlines potential specific actions that the data subject is approving. This does not refer to disclosing information. It is about what matters, proceeding and particular acts that are being consented to. In general, consent models, such as the one proposed by the Swiss Academy of Medical Sciences often include sentences like ‘I herewith agree that my health-related data and samples collected during health care (ambulant or as an inpatient) will be available for research purposes’ [33]. This phrase does not say much about what the person is approving to and therefore what action will consequently take place and call into question if this consent is equally valid for simple procedure, as well as, for high-risk research too. By laying out the specific matter, we better define the action that is taking place.

### Benefits

It is the core responsibility of a researcher to clarify to the data subjects the difference between receiving care or any treatment and enrolment in research beyond overcoming the risk of therapeutic misconceptions [3]. There is a strong belief in the good will and altruism that drives individuals to participate in research [34]. Thus, benefits often occur in various forms, from receiving tailored information to monetary profit. When profit is received, participants shall be made aware that there might be the possibility of commercial exploitation of their data in addition to a clear outline of any philanthropic benefits. This might change the nature of the action and the terms of the agreement, and thus of the consent given.

### Timeframes

Consent has a timeframe meaning the act of consent might change over time. In contrast to what sometimes seems assumed, consent is not reversible; rather, it is revocable. Revoking consent is different from withdrawing consent, it puts a stop to consent rather than cancels the consent: the consent was still valid and existing until it was revoked. Revoking consent is thus part of the consent continuum and it has some complex operational implications. From the point of revoking consent, positive consent is no longer in effect. When informed consent occurs during the face-to-face or any live interaction between a researcher and the potential participant, the participant can say “yes” and then change his or her mind and revoke consent; this spoken word fully and effectively revokes consent. This revokes the effect by simply announcing that we no longer wish to participate and this is often enough to erase the approval, and is usually thought to undo consent. However, rather than a true undoing, this revocation actually defines the end of the period during which consent existed. It is not retroactive; research that was conducted while consent existed is not deprived of it by the revocation. Continuation of such research, or any future project, is, however, unconsented from that point onwards. This clarifies some of the difficulties associated with revocation of consent. Data may have already been used and this cannot be undone, data sets might already be fully anonymized which render individual data impossible to retract. Furthermore, there may sometimes be formal institutional procedures for revoking consent. Even if we express our revocation of consent, it may take some time to be effective. As the data and the decision we make about it may outlive us, it is important to consider the timeframes of our approvals. For deceptive research allowed by ethics committees research and legislation in place, consent will be a retroactive act.

### Research goals

Altruism, goodwill, and contribution to the public good are often motivations to participate in research. Research that aims to generate public well-being tends to gain more support than research that envisions profit, despite addressing perhaps a well needed scientific vacuum [26]. The generation of profit is a very contentious issue. Although profit may be seen differently if reinvested to public services and ultimately generates a public good. The legal literature highlights issues in which the sample donated by an individual due to its special characteristics could lead to a scientific and commercial breakthrough by the development of a drug or a treatment [35]. This issue will be treated differently across jurisdictions but despite the particularities of each judicial system, data self-determination is a right. The goal of the research does change the nature of participation and the conditions under which an individual shall consent.

### Risk

Deciding on the level of risk is a key part of enhancing the data subject’s control over effective authorisation. It will not be possible to clearly and intelligibly outline all risk related to each data use for today or the future. However, Levels and or categories tend to be laid out in domestic legislation, ethical guidelines and ethics literature. These frameworks counterbalance the subjectivity of the perception of risk. It establishes a categorisation that could guide future assessments.

### Jurisdictions

Informed consent is a right protected by various domestic and international frameworks [2]. Professional codes, statutes, and administrative regulations change across jurisdictions. Data subjects must invest a significant degree of trust in those who will manage and use their data. I can share my data with a governance structure I trust and abide by rules I deem appropriate. But that may change over time, or the data may travel to other jurisdictions that may even put us at risk. It is crucial that the data subject can anticipate the level of protection desired. This action warrants protection to data subject and responsibilities to research agents. Protection and mechanism that provide compensation are guaranteed by chosen jurisdiction.

### Research agents

We do not present an exhaustive list of research agents, thus we name those often present in biomedical research. Rivas Velarde et al. [36]. claimed that people were likely to donate their data for research if the research actors are perceived to be professionally competent, generating public good, and being under effective governance. They explain that research competence, if it does not generate public good or is not regulated, erodes trust in research actors, and as a result, people are less likely to consent to participate in research. Whether your data may end up on an open access server or on highly secure data storage and who will use and have access to it changes the consent dynamics. This action allows data subjects or legal representatives to clearly select who is granted consent. Therefore, the moral and oftentimes legal responsibility is clearly deposited in a research agent(s) that will be accountable to a jurisdiction.

This periodic table of consent identifies elements already known, and we hope they can be used to discover new elements, characteristics, and dynamics. Researchers can use the data in the table to figure out how the new elements may behave or which elements the new elements may be similar through this comparison.

### Summary of the framework

This framework clearly outlines the elements involved in the act of consent. It provides a rationale for the choices and decisions presented to the potential research participant. We argue that there are benefits to enhancing disclosure and transparency and to creating a meaningful discussion between research participants and research agents is advantageous. It shall help to address ambiguities in highly contentious areas, such as instances in which profit is generated by research. By laying out this choice, there is enhanced clarity on the agreement. It will make it easy to identify potentially abusive set-ups where there is an uneven distribution of the benefits and burden of research. It will facilitate the continuation of research studies where researchers rotate, change focus, or discover new areas of investigation in their data. It will provide a platform for individuals and researchers to define what is reasonably achievable by participating in research, and what they consent to. We envision that this framework could be implemented both on traditional face-to-face consent procedures or paper-and-pencil formats as well as on web-mediated platforms. In all cases, it shall enable more transparent and trustworthy acts of consent.

Let’s look at an example. Traditional consent is informed consent either in person or via digital platforms. Informed consent procedures shall require the actor requesting the consent to (a) disclose relevant information for potential participants to judge risk and benefits. Actors may not foresee future use of data, or the consequences of the data re-use, and could therefore will be unable to comply with this requirement; this is true both in person and in the digital space. Then, the data subject will be assessed on whether there was a (b) effective comprehension of this information by the research subject. This is already highly complex in face-to-face interaction and nearly unattainable in the digital world. In the following step, (c) voluntariness, the choice to give or withhold consent must be freely made, and nudges on the page’s design and other functionalities are considered to as weakening respect for autonomy. (d) Competence refers to decision-making capacity regarding the presented choice; the assessment of competence or lack of it is particularly problematic, can be discriminatory in its implementation, and is often not feasible [24]. The final element, (e) consent, is often present as a static action rather than a continuous process. As we have seen, such as view is problematic, if a persons decide to stop their initial authorisation on digital or analogue system, the execution of this termination may take some time to be put in place. Consent is not static. When consent is withdrawed, it put an stop to an authorisation but does not invalidate the timeframe during which your consent was valid. Furthermore, a major flaw in traditional view of consent procedure is thus the little attention given to the re-use of data, change of actors and timeframes.

Our framework, and the table that illustrates its implementation, aim to address such shortcoming. We would argue that a consent process based on the use of our table would enable data subjects to choose to consent to agents whom they trust and deny consent to others in the present or the future. They will know that data will be shared, re-used only for some research purposes that they have consent to and not others today and in the future. If they change their mind, they can update their preference knowing that prior use of data was legitimate. The framework does not fully address issues with competence or incompetence, thus allowing attention to this category to call for the use of relevant frameworks such as non-discrimination legislation. Lastly, the choice provide aim to enhance transparent and trustworthy acts, not only to enlarge choices to all options possible. Using this approach would clarify who is allowed to do what with data, and thus enable data subjects to evaluate whether agents are likely to be competent, reliable, and honest. Without such clarity, this evaluation is impossible. Our approach could thus enhances the trustworthiness of agents and with it the bases of trust that consent acts require.

### Limitations of the framework

This framework is not exhaustive. There are elements and interactions that we may have overlooked. We have presented it as a tool to be further developed and tested. It is intended to present informed consent as a compositional act - a bundle of actions accomplished in the act of speaking, writing, or clicking one’s consent. There may be uses or research agents that do not yet exist; we anticipate that this will open up a dialogue in the future and that it will be built upon. Furthermore, some may see this framework as too rigid. It does make the choices and acts of consent explicit and specific, and thus perhaps rigid. This framework could be envisioned as feeding an opt-out model that relies on research participants not refusing consent. It is, therefore beyond the scope of this paper to consider the issues that will emerge from such an approach.

Our approach to consent as an act that is not retractable but revocable may be objected to, as it implies that the individual is not the master of all their personal information and material and could be viewed as challenging the individual’s personal autonomy. This understanding of consent, however, tracks the reality of data research more truthfully than a view in which consent can be cancelled outright. Once data is donated, used, and published, consent cannot be retroactively undone; once data is fully anonymised it cannot be tracked back and pulled out from data sets. Therefore, retracting consent in such a scenario is not possible. Moreover, viewing consent as retractable enables transparency. Researcher shall use data while consent is valid. Not having a clear timeframe would open questions about data was use legitimately, for example if a persons withdraw their consent, those who used their data before withdraw will be respecting their choice, as those who did not use the data after the withdraw.

Another objection might emerge depending on how the framework is implemented. Perhaps health personnel carrying out consent procedures and data scientists developing or managing informed consent platforms and databases might find our recommendations either too rigid or need further instructions. Implementation may also unveil potential burdens on the use of this framework. Thus, this is another step in the development of this framework, as it involves assessing the validity of consent against the traditional criteria set up by Beauchamp and Childress [3] or others. Our aim is to make explicit what is done or undone when consenting so that the bases of trust that consent acts require in biomedical science are enhanced.

### Concluding remarks

This paper focuses on what consent is and what it does or undoes. We present a framework that explores the basic elements of consent and breaks it down into its components. To conceptualize consent as a composition that results from the sum of its basic elements, we propose a new understanding of the informed consent mechanism that moves away from theorizing about what informed consent is, ought to be or assessing validation of a process in which consent is an endpoint. This model goes beyond only providing choices to potential research participants, it explains the rationale of those choices or consenting acts that take place when one speaks or writes an authorization to do something to someone. Furthermore, it contextualises consent acts in contemporary data-intensive biomedical research. It clarifies the retention and use of data in research. This table should enable exploring new elements, characteristics, and dynamics of informed consent. It is intended to foster meaningful discussions between research participants and research agents by outlining the specific matter and consent acts that take place. By clearly outlining the data subjects or legal representatives providing the authorisation of consent, the specific issue being consented and the specific agent seeking the consent(s), we aim to enhance the possibility to assess trustworthiness. The framework would allow data subjects to judge whether a specific agent is performing a specific act in a competent, reliable, and honest manner. Those seeking consent have a moral duty to be truthful about their intentions and actions. We argue that it is by clearly differentiating the goals and procedures of implementation, as well as what is done or undone when consent is obtained, that the bases of trust that consent acts require in the biomedical sciences can be enhanced.

## Data Availability

All data generated or analysed during this study are included in the manuscript.
